# Genetic Polymorphism of *GSTP1, GSTM1* and *GSTT1* Genes and Susceptibility to Chronic Myeloid Leukaemia

**DOI:** 10.31557/APJCP.2020.21.2.499

**Published:** 2020

**Authors:** Hadeil ME Idris, Abozer Y. Elderdery, Hiba B Khalil, Jeremy Mills

**Affiliations:** 1 *Department of Hematology, Faculty of Medical Laboratory Sciences, Al Neelain University, Sudan, *; 2 *Clinical Laboratory Sciences, Faculty of Applied Medical Sciences, Shaqra University, *; 3 *Clinical Laboratory Sciences, Faculty of Applied Medical Sciences, Jouf University, Saudi Arabia, *; 4 *School of Pharmacy and Biomedical Sciences, University of Portsmouth, UK. *

**Keywords:** GSTM1, GSTT1, GSTP1, Polymorphisms, CML

## Abstract

**Background:**

The development of cancer results from an imbalance between exposure to carcinogens and the capacity of various enzyme systems engaged in activation or in the detoxification of xenobiotics. The aim of the present study is to investigate the association of *GSTP1, GSTM1* and *GSTT1 *gene polymorphisms in susceptibility to Chronic Myeloid Leukaemia (CML).

**Methods:**

A total of 200 CML patients and 100 controls were enrolled in a case-control study with *GSTM1* and *GSTT1* analysis with PCR and *GSTP1* analysis with PCR-RFLP.

**Results:**

The *GSTT1* null genotype was significantly higher among CML patients suggesting that this genotype is associated with an increased risk of CML. It was found in 42% of cases as compared with 21% of the controls, (OR =2.78, 95% CI: 1.59 - 4.85; p-value =0.000). The presence of the *GSTT1* genotype may thus be considered a protective factor for CML. The frequency of individuals carrying GSTM1 null genotype was slightly higher in the control group but this difference was not statistically significant. The *GSTM1* null genotype was present in 35% of control cases and 34% of the CML patients, (OR=0.975, 95%CI: 0.58-1.58;p-value=0.863). Individuals with a combined *GSTM1* null/*GSTT1*null genotype had an estimated 2.85-fold increased risk of CML, but no associated risk between *GSTP1* Ile 105 Val polymorphism and CML was found (OR=1.99, 95% CI: 0.40 - 9.32; p-value = 0.417).

**Conclusions:**

No association between *GSTP1 *and *GSTM1* with susceptibility to CML was found. *GSTT1* genotype may be a protective factor for CML, while the null genotype shows association with developing CML.

## Introduction

Chronic Myeloid Leukaemia (CML) is a myeloproliferative disorder but a definite mechanism leading to this carcinogenesis is not yet completely understood (Elharam Ibrahim Abd allah, 2017). It is known that environmental exposure to cytotoxic and genotoxic agents derived from benzene may be associated with increased risk of CML (Bajpai et al., 2007). And genetic susceptibility studies of CML may serve to identify populations at risk here, together with its important disease mechanisms (Elharam Ibrahim Abd allah, 2017).

The development of cancer results from an imbalance between exposure to carcinogens (endogenous and exogenous) and the capacity of various enzyme systems engaged in the activation or detoxification of Xenobiotics. Inter-individual genetic variation in xenobiotic metabolizing enzymes has been associated with cancer development, (Kassogue et al., 2015) and since such a metabolism constitutes an important line of defense against a variety of carcinogens, inherited differences in the capacity of these enzymes may be an important genetic factor in cancer susceptibility (Ana Luisa, 2013). 

Xenobiotic metabolizing enzymes (XMEs) constitute one of the first lines of defence and they play a central role in the metabolism, elimination, and detoxification of xenobiotics or exogenous compounds introduced to the body (Omiecinski et al., 2011). Enzymes within the multiple enzyme system are classified in two categories namely Phase I and Phase II. The latter constitutes the theme of this study and its major detoxifying enzymes are Glutathione S-transferases (GSTs) which enable a wide variety of functions. GSTs fall into two distinct super-families: membrane bound microsomal GSTs and the soluble or cytosolic GSTs. Some genes of cytosolic enzymes play a crucial role in the detoxification of activated carcinogens and implications in cancer progress, particularly *GSTP1, GSTM1, *and *GST1* (Sailaja et al., 2010). Polymorphisms in these genes lead to the absence or decreased detoxification ability of enzymes, their dysfunction, and finally may impact on the risk of cancer development and heterogeneous drug responsiveness (Rostami et al., 2019). 

*GSTP1* gene possesses two variations in coding region, an A-to-G transition at105 codon and a C-to-T transition at 114 codon (Sailaja et al., 2010). The *GSTP1* Ile105Val polymorphism at nucleotide 313 in exon 5 of *GSTP1* lead to an amino acid substitution of isoleucine (Ile) by valine (Val) at amino acid position 105 (Ile105Val) (Dunna et al., 2012). This substitution potentially diminished the ability to detoxify certain carcinogens which would induce DNA adducts, ultimately leads to carcinogenesis (Sailaja et al., 2010). The frequency of the homozygous mutant genotype (Val/Val) ranged from 4% to 16% among White, 4% to 5% among Asian, and 19% for African-American ethnicities (Coughlin and Hall, 2002).

*GSTM1* polymorphism has been identified with three alleles; GSTM1*0, GSTM1*A and GSTM1*B (Engel et al., 2002). The former is a null allele consisting of the complete deletion of the *GSTM1* gene. Individuals who are homozygous for this allele are unable to produce the *GSTM1 *protein. The null variant of *GSTΜ1* is of particular interest as a plethora of studies have demonstrated the difference in its susceptibility, exposure to environmental toxicants, and resistance to chemotherapy treatment (Rostami et al., 2019; Mondal et al., 2005; Bhat et al., 2012). The frequency of the* GSTM1* null genotype ranges from 23% to 48% in African populations, 33% to 63% in Asian populations, 39% to 62% in European populations, and 23% to 62% in U.S. populations (Coughlin and Hall, 2002). 

The *GSTT1* gene is polymorphic and has two alleles, the GSTT1*1 and the GSTT1*0 (Mir Muhammad NasirUddin, 2014). The former is an active allele, having a crucial role in the Phase-II biotransformation of a number of drugs and industrial chemicals. The latter is a non-functional allele arising from the deletion of the *GSTT1 *gene, with null *GSTT1* (0/0) phenotype individuals being unable to form the GSTT1 protein (Bajpai et al., 2007). The homozygous *GSTT1* null phenotype has been described in different populations and shows wide variation (Bajpai et al., 2007; Rostami et al., 2019; Lordelo et al., 2012). The frequency of the *GSTT1* null genotype has been described in different ethnic groups with varying degrees as follows: 22% to 29% in African-Americans, 16% to 64% in Asian individuals, 15% to 27% in whites, 10% to 21% in European populations and 10% to 12% in Mexican-Americans (Coughlin and Hall, 2002). Therefore these alleles and the previous ones have taken a place in this study. 

The association between *GSTP1* Ile105Val, *GSTM1 *and *GSTT1* polymorphisms in the susceptibility to CML was investigated by different studies, but with conflicting results (Banescu et al., 2014; Lordelo et al., 2012; Al-Achkar et al., 2014; Lourenco et al., 2005; Weich et al., 2016; Hishida et al., 2005; Bhat et al., 2012). Therefore, this study aimed to determine the frequency of genetic polymorphism in GST (P1, M1 and T1) genes and to ascertain their association with CML within the Sudanese population.

## Materials and Methods

This cross-sectional, case-control study was conducted at the Radiation and Isotopes Center of Khartoum, (RICK). The study included 200 patients diagnosed with CML, 68 (34%) females and 132 (66%) males plus 100 age matched healthy controls, 49 (49%) females and 51 (51%) males; 300 participants in total. All CML patients were in chronic phase with exception of one patient who was in an accelerated phase.

The Institutional Ethical Committee of Al-Neelain University approved the study and written consent was obtained from all the participants involved. Patients suffering from any other disease such as chronic myelomonocytic leukaemia and other myeloproliferative disorders were excluded. The control group consisted of healthy unrelated volunteers without a medical history of cancer and all patients and controls were of Sudanese ethnicity.

DNA extraction: Venous blood samples (3ml) were collected into EDTA containing vacutainers. Genomic DNA used for polymorphic analysis was extracted by guanidine chloride method and isolated DNA was stored in Tris EDTA buffer at -20°C till use.

GST P1 polymorphism: The genetic polymorphism analysis for GSTP1was determined by using the polymerase chain reaction/ restriction fragment length polymorphism (PCR/ RFLP) method. Primer pairs used were synthesized from Macrogen) F5′-ACCCCAGGGCTCTATGGGAA-3′ and R5′TGAGGGCACAAGAAGCCCCT-3′). 

A three-step PCR was standardized using Sensoquest thermocycler, involving initial denaturation at 95°C for 3 min, followed by 35 cycles of denaturation at 94°C for 30 sec, annealing at 61°C for 30 sec, and extension at 72°C for 40sec – with a final extension at 72°C for 5 min.

Amplification products corresponding to 176bp were then visualized after electrophoresis in an ethidium-bromide-stained 2% agarose gel and then subjected to restriction enzyme analysis with BsmA1 (New England BioLabsInc). 

Quantities 0.2 μl of BsmA1enzyme, 2 μl of enzyme buffer, 5 μl of the PCR product and 2.8 μl of double distilled water were then incubated overnight at 37°C. 

Three banding patterns were observed on 3% agarose gel stained with ethidium-bromide and these were; 176 bp bands corresponding to the AA (Ile/Ile, homozygous wild type genotype), 176, 91 and 85 bp bands corresponded to the AG (Ile/Val, heterozygous genotype) and 91, 85 bp bands that corresponded to the GG (Val/Val, homozygous mutant genotype).

*GSTM1* and *GSTT1* polymorphism: The polymorphic deletion of *GSTM1* and *GSTT1 *genes were genotyped using the multiplex PCR approach. Primers used for *GSTM1* and *GSTT1* amplification were synthesized from Macrogen: F5’-GAA CTC CCT GAA AAG CTA AAG C-3’; R5’-GTT GGG CTC AAA TAT ACG GTG G-3’ and F5’-TTC CTT ACT GGT CCT CAC ATC TC-3’, R5’-TCA CCG GAT CAT GGC CAG CA-3’, respectively. An 268-bp fragment of β-globin gene amplified by F5’-CAA CTT CAT CCA CGT TCA CC-3’, R5’- GAA GAG CCA AGG ACA GGT AC-3’ primers was used as internal positive control. 

The thermocycling procedure (Techne GeniusTC-412 Thermal Cycler), involved initial denaturation at 94°C for 4 minutes, followed by 35 cycles of 1 minute at 94 °C, 45 seconds at 55°C, 1 minute at 72°C and a final extension for 10 minutes at 72°C. Genotyping of the genes (null genotypes) is revealed by the absence of the 219 bp for *GSTM1* and 480 bp for *GSTT1* PCR products. PCR products for the genotyping of polymorphisms were visualized by 2% agarose gel electrophoresis with ethidium bromide. The absence of β-globin amplification here indicated a failure of PCR reaction. 

Statistical analysis: Statistical analysis included descriptive statistics of the mean using standard deviation. The Odds ratio (OR) with a confidence interval (CI) of 95% was calculated by logistic regression. Pearson’s chi-square test was then used to compare genotype distribution between patients and control, with p-values less than 0.05 being considered as statistically significant. 

## Results

The distribution of the *GSTP1, GSTM1* and *GSTT1 *genotypes in CML patients and controls are shown in Table 1. The homozygous (Val/Val) of GSTP1, the heterozygous (Ile/Val) and the wild genotype of GSTP1 (Ile/Ile) forms were found in 3.5%, 31.5% and 65% of CML cases, respectively.

In the Control, the homozygous (Val/Val) of *GSTP1* Ile105Val, heterozygous (Ile/Val) and the wild genotype of *GSTP1* (Ile/Ile) forms were 2%, 36% and 62%, respectively. The homozygous mutant type (Val/Val) showed no significant difference between patients and controls (OR=1.992, 95% CI: 0.396-9.322; P-value = 0.417).

The frequency of individuals carrying the GSTM1 in patients and controls were 44% and 21.7% respectively. 

The *GSTM1 *null genotype frequency was found to be slightly higher in the control group, (35% as opposed to 34% in CML patients), but this difference was not considered to be statistically significant (OR =0.975, 95% CI: 0.578-1.584; p- value = 0.863).

*GSTT1 *was found in 57.5% of CML patients and 79% of the Control but the frequency of individuals carrying the *GSTT1* null genotype was significantly higher among CML patients, 42% compared to 21% of the Control; (OR =2.781, 95% CI: 1.593-4.853; p-value =0.000). The *GSTT1* genotype presence may thus be considered a protective factor for CML.

The combined effects of *GSTM1* and *GSTT1 *genotypes in CML risk were also conducted. Individuals with a combined *GSTM1* null/*GSTT1* null genotype had an estimated 2.847-fold increased risk of CML over individuals with a *GSTM1* present /*GSTT1 *present genotype (OR=2.847; CI=1.288-6.293; p-value=0.000). In contrast, the *GSTM1* present /*GSTT1* null and *GSTM1 *null/*GSTT1* present genotypes were not associated with a CML risk (p-value=0.064 and 0.061 respectively), see Table 2.

**Table 1:** Distribution of *GSTP1, GSTM1* and *GSTT1* Genotypes in CML Patients and Control

Genotypes/ Allele frequency		CML N (%)	Control N (%)	OR	95%CI	P-value
*GSTP1*	AA	130 (65)	62 (62)	Reference		
	AG	63 (31.5) ^(b)^	36 (36)	0.84	0.51 - 1.40	0.505
	GG	7 (3.5) ^(b) ^	2 (2)	1.99	0.40 - 9.32	0.417
	A	323 (80.8)	160 (80)	Reference		
	G	77 (19.3%)^(b)^	40 (20%)	1.05	0.68 - 1.61	0.827
*GSTM1*	Present	132 (66)	65(65)	Reference		
	Null	68 (34) ^(b)^	35(35)	0.98	0.58 - 1.58	0.863
*GSTT1*	Present	115 (57.5)	79(79)	Reference		
	Null	85 (42) ^(a)^	21(21)	2.781	1.59 - 4.85	0

N, total number; OR, odd ratio; CI, confidence interval; Statistical significance (P-value) is shown in superscript parenthesis; ^(a)^, <0.05; ^(b)^, >0.05

**Table 2:** Combination Effect of *GSTM1* and *GSTT1* Genotypes on CML Risk

Genotypes		CML N (%)	Control N (%)	OR	95%CI	P-value
*GSTM1*	*GSTT1*					
Present	Present	91 (45.5)	53 (53)	Reference		
Present	Null	41 (20.5) ^(b)^	12 (12)	1.99	0.96 - 4.12	0.064
Null	Present	24 (12) ^(b)^	26 (26)	0.54	0.28 - 1.03	0.061
Null	Null	44 (22) ^(a)^	9 (9)	2.85	1.29 - 6.29	0

N, total number; OR, odd ratio; CI, confidence interval; Statistical significance (P- value) is shown in superscript parenthesis; ^(a)^, <0.05; ^(b)^, >0.05.

## Discussion

Several previous studies have reported an association between the polymorphisms of the GSTs genes and the susceptibility of developing certain types of cancer. Such cancers include lung cancer (Mir Muhammad NasirUddin, 2014), ovarian cancer (Coughlin and Hall, 2002) and breast cancer (Hashemi et al., 2012). Previous studies have reported about GSTs association with heamatological malignancy, but with conflicting findings as mentioned previously (Dunna et al., 2012; Zhou et al., 2013; Rostami et al., 2019; Weich et al., 2016). In the present study, 3.5% of CML patients and 2% of the control had the homozygous type (Val/Val) of *GSTP1* Ile105Val polymorphism. No significant difference was found between patients and the control and the study also revealed no association between *GSTP1* Ile 105 Val polymorphism and the risk of developing CML:(OR=1.992, 95% CI: 0.396-9.322; p-value = 0.417). 

This finding was in agreement with a study conducted in Turkey by Karkucak et al who found no evidence between *GSTP1*Ile105Val and CML susceptibility (p-value 0.199) (Mutlu Karkucak, 2012). Weich and co-workers also reported no meaningful association between *GSTP1*Ile105Val and CML risk (Weich et al., 2016). Several studies disagree with our findings in stating that there may be a relationship between *GSTP1 *Ile 105 Val polymorphism and CML development/prognosis (Sailaja et al., 2010; Rostami et al., 2019; Banescu et al., 2014). Some of these studies relied on smaller sample size and others on very broad confidence levels. Such contrary findings may also not have taken into account the geographical and ethnic differences in the type of environmental carcinogens samples the patients may have been exposed to (Sailaja et al., 2010; Banescu et al., 2014).

Homozygotes for the null allele (deletion) of *GSTM1 *and *GSTT1* lack the activity of respective enzymes. This makes the reactivity of electrophilic substrates low and therefore affects the function within cellular macromolecules. (Hashemi et al., 2012). The* GSTM1* null and *GSTT1* null appear to be associated with a significant risk of several types of cancers, such as hematological neoplasm (Banescu et al., 2014; Sailaja et al., 2010; Zhou et al., 2013) and breast cancer (Hashemi et al., 2012). It has been shown that the risk of ALL is doubled in patients who carry the *GSTM1* deletion (Joseph et al., 2004). The *GSTT1* null genotype was also demonstrated as a risk factor for both AML and ALL in some ethnic groups (Zhou et al., 2013).

Interestingly, no association between *GSTM1* null genotype and the risk of CML was found here: (OR =0.975, 95% CI: 0.578-1.584; p- value = 0.863). However, Muddathir and his co-workers found that *GSTM1* null genotype was associated with increased risk of CML among Sudanese(Muddathir et al., 2019). This is not unexpected because Sudan is multiethnic with an admixture of Arab and African lineages which may be associated with differences in genetic makeup. The tribal differences within the participant populations in these two studies, irrespective of different in sample sizes, may contribute to this variation in the results. In contrast, Taspinar et al and Hishida et al claimed that *GSTM1* polymorphism was not associated with the risk of CML, which was in agreement with our study (Hishida et al., 2005; Taspinar et al., 2008). Al-Achkar and co-workers however, concluded that *GSTM1 *null genotype was associated with increase risk of CML: (OR=2.55; 95% CI; 1.54-4.22; p-value = 0.0002) (Al-Achkar et al., 2014). An interesting result was observed by Lordelo et al who found a positive association between CML risk with *GSTM1* present genotype and that the *GSTM1* null genotype decreased this risk (Lordelo et al., 2012). 

In the present study, we observed that the frequency of *GSTT1* null genotype in CML patients was significantly higher than in the control (OR =2.781, 95% CI: 1.593 - 4.853; p-value =0.000). *GSTT1* null genotype frequency posed a 2.781 fold increase in the risk of CML, compared to those possessing both alleles. Therefore, the *GSTT1 *genotype may be a protective factor for CML, whilst the null genotype showed an association with the development of CML. The above findings were in agreement with a recent study conducted in Sudan (Muddathir et al., 2019). Many studies have focused on the relationship between *GSTT1* polymorphism and the risk of CML in diverse ethnic groups (Bajpai et al., 2007; Al-Achkar et al., 2014; Kassogue et al., 2015). These findings were in agreement with this study. In contrast however, a Japanese study reported no significant association between *GSTT1 *and the risk of CML (OR=1.32, 95% CI; 0.74-2.36; p-value= 0.353) (Hishida et al., 2005). 

*GSTT1* is involved in the metabolism of ethylene oxide (EO) - a genotoxic agent capable of producing heritable translocations and increasing the frequency of spontaneous chromosome abnormality. Presuming that EO is higher in *GSTT1* null individuals, it was hypothesized that the loss of the *GSTT1* gene may be an influencing factor in the production of the Philadelphia chromosome (Ph) associated with CML (Lourenco et al., 2005). 

A combined analysis was conducted to assess the role of polymorphic variants on CML risk. We observed a significant interaction between the *GSTM1* null and *GSTT1* null genotypes, and thus, individuals carrying the null genotype of both are at a higher risk to CML: (OR=2.847; CI=1.288-6.293; p-value=0.000). It might be inferred from the data that the both genes (*GSTM1* and *GSTT1*), act in a synergistic way and are important to the detoxification system (Bhat et al., 2012). When these genes lack their enzyme activity and become inactive there is an increased opportunity for DNA damage, resulting in the risk elevation of the double null genotype to CML (Ozten et al., 2012), as found in this study. The risk elevation of this double null genotypes was also in accordance with the Al-Achkar and co-worker study (Al-Achkar et al., 2014). Taspinar et al., (2008) also suggested that the association between the *GSTT1 *or *GSTM1 *genotype and CML depends on relative expression levels. Therefore the current result suggested a distinguished haplotype which was found with a higher susceptibility in having CML as detected in not many ethnic groups.

In the present study, it was not possible to compare GSTs polymorphism within the clinical phases of CML, because only one sample was found to be on accelerating phase with no blast crisis. However, A study has reported significant increases in the frequency of the *GSTP1* mutant allele Val in the advanced disease state (accelerated and blast crises), as compared to the chronic phase (Sailaja et al., 2010). Furthermore, Lourenco et al reported the frequency of the *GSTM1* null genotype being lower in patients in the accelerated phase or with blast crisis than those patients in the chronic phase:(20.0% vs. 49.0%, p-value 0.01) (Lourenco et al., 2005). In contrary, Bənescu et al., (2014) found no association between *GSTM1 *null or* GSTT1 *null and the clinical phases of CML. 

In conclusion, this study found that the percentage of the *GSTT1 *null genotype in CML patients was significantly higher than in the control and thus the *GSTT1 *genotype may be assumed a protective factor for CML, with the null genotype associated with the development of this disease. Additionally, no association between *GSTP1 *and *GSTM1* and the susceptibility to CML was found. This study may provide a basis for further more extensive testing in a larger Sudanese population.
